# Occupational characterization of workers exposed to asbestos: an integrative review

**DOI:** 10.47626/1679-4435-2022-733

**Published:** 2023-02-13

**Authors:** Kauane Vicari, Inaye Mayr Ribeiro, Bianca Fontana Aguiar, Christiane Brey, Shirley Boller, Fernanda Moura D’Almeida Miranda

**Affiliations:** 1 Departamento de Enfermagem, Universidade Federal do Paraná, Curitiba, PR, Brazil

**Keywords:** asbestos, occupations, occupational exposure, occupational health, asbesto, ocupações, exposição ocupacional, saúde do trabalhador

## Abstract

Asbestos is a mineral fiber abundant in nature and classified as a carcinogen since 1987. The present study aimed to identify, in the scientific literature, what are the occupation and activities developed by sick workers and which categories would be affected with asbestos-related diseases. Through a literature review performed in the following databases: PubMed, CINAHL (Cumulative Index to Nursing and Allied Health Literature), Web of Science, and Regional Portal of the Virtual Health Library, 23 studies published from 2015 to 2020 were selected and evaluated. The occupations that showed greater illness due to exposure to asbestos were general asbestos workers (40%), miners (22%), and textile workers (9%), followed by naval, automotive, carpentry, doll-making, construction, and upholstery workers, as well as workers involved in the rescue, recovery, cleaning, and restoration of the World Trade Center (4%). Of the disease associated with exposure to asbestos, the most described is malignant mesothelioma (43%). Evidence found corroborate pre-existing information in the literature showing that exposure to asbestos may be harmful to health. Moreover, the importance of using personal protective equipment was emphasized, in order to prevent the development of asbestos-related diseases.

## Abstract (pt)

O asbesto é uma fibra mineral abundante na natureza, classificado como substância cancerígena desde 1987. Objetivou-se identificar, na literatura científica, quais são as ocupações e atividades desenvolvidas por trabalhadores adoecidos e quais categorias seriam afetadas com doenças relacionadas ao asbesto. O presente estudo trata-se de uma revisão de literatura realizada mediante a busca nas bases de dados PubMed, CINAHL (Cumulative Index to Nursing and Allied Health Literature), Web of Science e Portal Regional da Biblioteca Virtual em Saúde, sendo selecionados e avaliados 23 estudos publicados entre 2015 e 2020. As ocupações que demonstraram maior adoecimento pela exposição ao asbesto foram trabalhadores gerais de indústrias de asbesto (40%), mineradores (22%) e do setor têxtil (9%), seguidos por trabalhadores da indústria naval, do setor automotivo, carpinteiros, do setor de fabricação de bonecas, da construção civil, estofadores e trabalhadores que participaram do resgate, recuperação, limpeza e restauração do local do World Trade Center (4%). Entre as patologias associadas à exposição ao asbesto, a principal descrita é o mesotelioma maligno (43%). As evidências encontradas corroboram as informações preexistentes na literatura de que a exposição ao asbesto pode ser prejudicial à saúde. Ainda, ressalta-se a importância da utilização de equipamentos de proteção individual, a fim de evitar o desenvolvimento de doenças relacionadas ao asbesto.

## INTRODUCTION

Asbestos is a collective term used for commercial identification of a heterogeneous group of minerals whose crystals form bundles of easily separable fibers. These minerals derive from eruptive metamorphic rocks that, due to a natural process of recrystallization, undergo changes, forming a fibrous material. They belong to two different groups: a) amphiboles, which includes amosite (brown asbestos), crocidolite (blue asbestos), anthophyllite, actinolite, and tremolite; and b) serpentines, represented by chrysotile (white asbestos).^1,2^

The applicability of this mineral goes beyond the construction industry. There are reports of more than 3 thousand products made of asbestos, such as water tanks, asbestos-cement tiles, canvas and brake pads for cars and trucks, fabrics, fireproof blankets, thermal insulation fabrics, vinyl flooring, hydraulic cardboards, automotive gaskets, inks, asphalt, and reinforced plastics.^1,3^

Surely, exposure to asbestos contributes to the development of asbestos-related disease (ARD), resulting from biopersistence of fibers in the lung.^4,5^ Furthermore, the José de Alencar Gomes da Silva National Cancer Institute (Instituto Nacional de Câncer José de Alencar Gomes da Silva, INCA) and the International Agency for Research on Cancer (IARC) state that the aforementioned fibers are potentially carcinogenic agents.^6^

With regard to ARDs, it is worth highlighting malignant mesothelioma (MM), lung neoplasms, and non-malignant conditions, such as asbestosis and development of pleural plaques.^7^ Occupational exposure is one of the main factors for the onset of ARDs and other diseases as well, since some workers are routinely exposed to different substances harmful to health.^8,9^

Due to the applicability of asbestos in several products, its use was widespread in some countries such as Russia, China, Brazil, Thailand, Kazakhstan, India, and Ukraine.^10^ Brazil also stood out as producer and exporter, a fact that deserves attention and monitoring by health services due to the possible occurrence of ARDs.^11,12^

Total ban of asbestos is already a reality in at least 66 countries,^13,14^ and, in Brazil, only in 2017 the Federal Supreme Court (Supremo Tribunal Federal, (STF) established the prohibition of extraction, manufacturing, and sale of chrysotile asbestos in the entire national territory.^15^

ARDs are often identified lately, due to their long period of latency, which lasts from exposure to the first clinical symptoms, thus contributing to disease underregistration.^16,17^ In light of the foregoing, knowledge of the possible at-risk groups and of the prevalent diseases resulting from occupational exposure has become indispensable. Based on these considerations, the aim of this study was to identify, in the scientific literature, what are the occupations and activities developed by sick workers and what categories are most affected with ARDs.

## METHODS

This was an integrative literature review, a method that allows for gathering and synthesizing evidence available in articles produced about a topic.^18^

This research was developed based on Whittemore & Knaff^19^, who proposed the following steps: 1) formulation of the research question; 2) data collection; 3) data assessment according to inclusion and exclusion criteria; 4) data analysis; and 5) presentation of results and knowledge synthesis.

The guiding research question was structured using the non-clinical research strategy characterized by the acronym formed by the words population/patient/problem, interest, and context (PIC), being P: workers; I: disease caused by asbestos; and C: exposure to asbestos. Therefore, the resulting question was “what characterizes workers with diseases resulting from exposure to asbestos?”.

Inclusion criteria consisted of studies published from January 2015 to January 2020 and written in English, Italian, Portuguese, or Spanish. Exclusion criteria consisted of duplicate articles and those not fully available online, as well as theses, dissertations, letters, and editorials from scientific journals. The search for studies was performed from February to March 2020, in the following databases: PubMed, CINAHL (Cumulative Index to Nursing and Allied Health Literature), Web of Science, and the Regional Portal of the Virtual Health Library (VHL), which gathered findings from the MEDLINE and IBECS databases. The following Health Science Descriptors (Descritores de Ciências da Saúde, DeCS) were used: “Workers”, “Asbestos”, “Amianto”, “Occupational exposure,” and “Inhalation exposure”.

**Figure 1:**
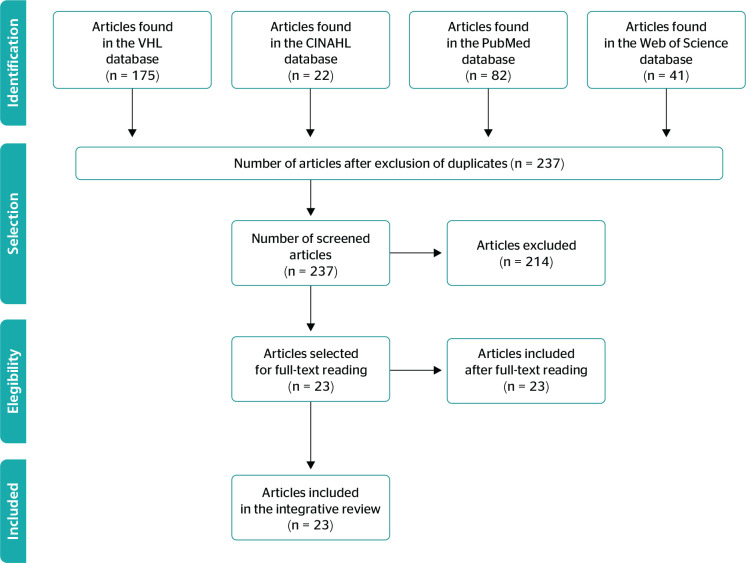
Study selection flowchart, adapted from the Preferred Reporting Items for Systematic reviews and Meta-Analyses (PRISMA) model, Brazil, 2020.

This search identified 320 studies, 175 in BVS, 22 in CINAHL, 82 in PubMed, and 41 in Web of Science, and, after application of exclusion criteria, 237 publications remained in the analysis. These publications were then analyzed after reading of abstracts, and, finally, 23 articles were considered eligible, which constituted the sample of this review. The study selection process was based on the recommendations of Preferred Reporting Items for Systematic reviews and Meta-Analyses (PRISMA),^20^ as shown in Figure 1.

CINAHL = Cumulative Index to Nursing and Allied Health Literature; VHL = Virtual Health Library.

## RESULTS

The final sample consisted of 23 articles that addressed the occupational relationships of workers with diseases resulting from exposure to asbestos. Of these articles, 17 (74%) were cohort studies, 5 (22%) were case studies, and 1 (4%) was a pilot study. With regard to year of publication, four of them were published in 2015, three in 2016, nine in 2017, five in 2018, and two in 2019.

Concerning the country where the study was conducted, Italy was the leading country, with seven (30%) articles, followed by the United States (USA), with five (22%); South Korea, China, and France, with two (9%) articles each; and then Germany, Belgium, Great Britain, Japan, and Spain, with one (4%) article each.

As for workers’ occupations reported in the studies, nine (40%) articles included general asbestos workers; five (22%), miners; two (9%), textile workers, followed by workers in the naval, automotive, carpentry, doll making, construction, upholstery industries, and workers who participated in the rescue, recovery, cleaning, and restoration of the World Trade Center, with one (4%) publication each.

Corning the study population, it was observed that, in 16 (70%) articles, sick workers were men; whereas four (17%) studies involved both men and women and three (13%) studies included only female participants.

Among the ARDs that affected workers, articles reported cases of MM in ten (43%) publications; lung cancer and other neoplasms, such as those affecting mouth, pharynx, esophagus, liver, bile duct, ovary, and colon, were found in eight (35%) publications; and respiratory diseases, asbestoses and the development of pleural plaques were observed in five (22%) studies.

Chart 1 presents the main characteristics of the articles selected for this review.

**Chart 1:** Characterization of the selected articles, Curitiba, Paraná, Brazil, 2020

Title	Authors	Language	Country	Productive industry	Research design	Journal and year of publication
1. “Cancer attributable to asbestos exposure in shipbreaking workers: a matched-cohort study”	Wu et al.^21^	English	China	Naval industry	Retrospective cohort	PLoS ONE, 2015
2. “Enduring health effects of asbestos use in Belgian industries: a record-linked cohort study of cause-specific mortality (2001-2009)”	Borre & Deboosere^22^	English	Belgium	General asbestos	Retrospective cohort	BMJ Open, 2015
3. “Malignant mesotheliomas in textile rag sorters”	Chelini et al.^23^	English	Italy	Textile industry	Retrospective cohort	Annals of Occupational Hygiene, 2015
4. “CT characteristics of pleural plaques related to occupational or environmental asbestos exposure from South Korean asbestos mines”	Kim et al.^24^	English	South Korea	Mining	Retrospective cohort	Korean Journal of Radiology, 2015
5. “Malignant mesothelioma in a motor vehicle mechanic: case report and review of the literature”	Meisenkothen^25^	English	United States	Automotive industry	Case study	NEW SOLUTIONS: A Journal of Environmental and Occupational Health Policy, 2016
6. “Pleural plaques and lung function in the Marysville worker cohort: a re-analysis”	Zu et al.^26^	English	United States	Mining	Retrospective cohort	Inhalation Toxicology, 2016
7. “Pleural mesothelioma: case-report of uncommon occupational asbestos exposure in a small furniture industry”	Oddone & Imbriani^27^	English	Italy	Carpentry	Case study	International Journal of Occupational Medicine and Environmental Health, 2016
8. “Asbestos-related lung cancers: a retrospective clinical and pathological study”	Ugues et al.^28^	English	France	General asbestos	Retrospective cohort	Molecular and Clinical Oncology, 2017
9. “Mortality of talc miners and millers from Val Chisone, Northern Italy: an updated cohort study”	Pira et al.^29^	English	Italy	Mining	Retrospective cohort	Journal of Occupational and Environmental Medicine, 2017
10. “Occupational asbestos exposure and incidence of colon and rectal cancers in French men: the Asbestos Related Diseases Cohort (ARDCo-Nut)”	Paris et al.^30^	English	France	General asbestos	Retrospective cohort	Environmental Health Perspectives, 2017
11. “Asbestos-related disease in upholsterers”	Cruz et al.^31^	English	Spain	Upholstery	Case study	Archives of Environmental & Occupational Health, 2017
12. “Mesoteliomi pleurici in addette alla fabbricazione di bambole: esposizione ad amianto?”	Barbieri et al.^32^	Italian	Italy	Doll making	Case study	La Medicina del Lavoro, 2017
13. “Mortality from asbestos-associated disease in Libby, Montana 1979-2011”	Naik et al.^33^	English	United States	Mining	Retrospective cohort	Journal of Exposure Science and Environmental Epidemiology, 2017
14. “Chest physician-reported, work-related, long-latency respiratory disease in Great Britain”	Carder et al.^34^	English	Great Britain	General asbestos	Retrospective cohort	European Respiratory Journal, 2017
15. “The effects of pleural plaques on longitudinal lung function in vermiculite miners of Libby, Montana”	Clark et al.^35^	English	United States	Mining	Retrospective cohort	The American Journal of the Medical Sciences, 2017
16. “Pleural and peritoneal mesotheliomas in the Friuli Venezia Giulia register: data analysis from 1995 to 2015 in Northeastern Italy”	D’Agostin et al.^36^	English	Italy	General asbestos	Retrospective cohort	Journal of Thoracic Disease, 2017
17. “Clustering of malignant pleural mesothelioma in asbestos factories: a subgroup analysis in a 29-year follow-up study to identify high-risk industries in Taiwan”	Lee et al.^37^	English	China	General asbestos	Retrospective cohort	BMJ Open, 2018
18. “Chest CT scan findings in World Trade Center workers”	Hoz et al.^38^	English	United States	Workers involved in the rescue, recovery, cleaning, and restoration of the World Trade Center	Retrospective cohort	Archives of Environmental & Occupational Health, 2018
19. “Cancer incidence in a cohort of asbestos-exposed workers undergoing health surveillance”	Barbiero et al.^39^	English	Italy	General asbestos	Retrospective cohort	International Archives of Occupational and Environmental Health, 2018
20. “Potential asbestos exposure among patients with primary lung cancer in Japan”	Tamura et al.^40^	English	Japan	General asbestos	Retrospective cohort	Journal of Occupational Health, 2018
21. “Ovarian cancer in a former asbestos textile factory worker: a case report”	Park et al.^41^	English	South Korea	Textile industry	Case study	Annals of Occupational and Environmental Medicine, 2018
22. “Investigating the association between occupational exposure to asbestos and ovarian carcinoma: results from a pilot study in Germany”	Rajput et al.^42^	English	Germany	General asbestos	Pilot study	BMC Public Health, 2019
23. “Malignant mesothelioma in construction workers: the Apulia regional mesothelioma register, Southern Italy”	Vimercati et al.^43^	English	Italy	Construction industry	Retrospective cohort	BMC Research Notes, 2019

## DISCUSSION

The selected studies revealed the hazards of asbestos and characterized workers with diseases resulting from exposure to this substance. Measures to restrict its use have already been implemented by several countries; however, its deleterious effects will last for decades in the health of many workers.^44,45^

ARDs are increasingly more common and are especially observed in regions where this mineral was widely employed, being also considered an occupational disease.^43,44,46^

Of the ARDs that affect the workers included in this research, it is worth noting the prevalence of MM, a rapidly evolving disease whose period of latency depends on time of exposure and may reach up to 50 years.^21,27,47,48^ A study conducted in Belgium in 2015 showed that mortality for MM was higher among blue-collar asbestos workers.^22^

It bears highlighting that, in other studies, workers of the following industries also presented with diagnosis of MM: naval, textile, automotive, chemical, metal, construction, and furniture.^21-23,25^ Furthermore, MM occurred among employees of a doll-making factory, whose exposure to asbestos has not been known so far.^32^

The incidence rates of MM were higher in the male population.^23,29,37^ The prevalence in men is justified by the occupations they perform, which often involve a direct handling of asbestos.^34,36^

Women diagnosed with MM were exposed to asbestos in the workplace, especially in the textile industry, or had a history of paraoccupacional exposure, probably derived from the household environment or from contact with a family member who worked directly with the mineral.^34,36,49^

Lung cancer was also identified, with incidence for workers of the naval industry^21,28,39^ and for those who handle asbestos directly.^22^ Trachea and bronchus cancer was also observed in this population.^21^

A relevant issue for the development of these diseases is the possible association between smoking and exposure to asbestos: the frequency of self-acknowledged smokers in a study was 79.5%,^31^ corroborating another research that found 74.4% of smokers with lung cancer.^41^ Therefore, smoking cessation is also necessary in the population exposed to asbestos.^31,50^ The synergy between smoking and exposure to asbestos potentiates the toxic effects of fibers and increase the risk for lung cancer.

Other neoplasms attributable to exposure to asbestos are those affecting mouth, pharynx, esophagus, liver, and bile duct, and mortality for these causes was found in blue-collar asbestos workers, followed by automotive, naval, and construction workers.^21,22^ This circumstance confirms the carcinogenic potential of the fibers.^51,52^

Contrary to the already reported types of cancer and in line with a study conducted in Italy, the occurrence of ovary cancer stands out in the female population, especially among women working in the textile industry.^41,42,53^ For the male population, in turn, findings point out to development of colorectal cancer.^30,54^

With regard to non-cancerous asbestos-related diseases, they include asbestosis and pleural plaques.^24,28^ Asbestosis is caused by the deposition of asbestos fibers in the lungs, leading to fibrosing interstitial pneumonitis.^55,56^ Pleural plaques represent a predisposing factor of ARDs.^38,40,55^

Miners are found to be the workers with the most extensive pleural plaques and asbestosis.^24^ In others studies, there are reports of the prevalence of these conditions in construction workers, followed by metal, mining, and furniture workers.^33,31,40^ This differs from findings reported in studies conducted in Marysville (United States) and in Libby (United States), which did not evidence an association between single pleural plaques and reduced lung function.^26,35^ However, the identification of these data, which are related to risk factors, help predict malignant comorbidities.^38,40^

Studies emphasize that workers exposed, directly or not, to asbestos should be informed about the hazards it causes, so as to provide better work conditions, as well as better health surveillance, for this population.^25,39,57^

It is important to reinforce the importance of characterizing workers, in order to promote health care measures aimed at those whose occupation is directly related to ARDs.

## CONCLUSIONS

Evidence found in this study makes it possible to characterize workers with diseases resulting from exposure to asbestos. Findings reveal that workers exposed, directly or not, to asbestos, are prone to the development of any type of ARD.

The main occupations of these workers consists of general asbestos workers, miners, carpenters, as well as those working in the textile, naval, and automotive industry. Associated diseases were MM, lung, ovary and colorectal neoplasms, and non-neoplastic conditions such as asbestosis and pleural plaques. In most studies, workers with diseases resulting from any type of ARD were men.

These data corroborate information showing that exposure to asbestos may be detrimental to health. The hazards of occupation exposure are highlighted; however, the risk is still imminent, since restriction of asbestos is not still a reality in many countries. Therefore, it is proposed to develop scientific productions and to implement effective health surveillance measures aimed at the most vulnerable population.

One limitation of this study was the non-inclusion of grey literature. However, the articles selected for this integrative review contributed to the outcome of the research, since they were recent articles published in international journal and addressing a relevant topic.

## References

[1] Castro H, Giannasi F, Novello C (2003). A luta pelo banimento do amianto nas Américas: uma
questão de saúde pública. Cien Saude Colet.

[2] Brum SC, Almeida B, Pelosi ES, Pacheco J, Guimarães MGA (2016). Amianto: a bioética entre o custo e a
toxicidade. Rev Eletronica Teccen.

[3] Alleman JE, Mossman BT (1997). Asbestos revisited. Sci American.

[4] Santos FRF, Souza Neto JA (2014). Identificação e quantificação de
amianto em solo no entorno de fábrica de materiais de
construção a base de fibrocimento, no bairro da Várzea,
Recife (PE). Estud Geol.

[5] Associação Brasileira dos Expostos ao Amianto (2020). Página inicial [Internet].

[6] Instituto Nacional de Câncer (2020). Amianto [Internet].

[7] Kratzke P, Kratzke RA (2018). Asbestos-related disease. J Radiol Nurs.

[8] Instituto Nacional do Câncer (2018). Exposição no trabalho e no ambiente - amianto
[Internet].

[9] Brey C, Gouveia FT, Silva BS, Sarquis LMM, Miranda FMA, Consonni D (2020). Câncer de pulmão relacionado à
exposição ocupacional: revisão
integrativa. Rev Gaucha Enferm.

[10] Marsili D, Terracini B, Santana VS, Ramos-Bonilla JP, Pasetto R, Mazzeo A (2016). Prevention of Asbestos-Related Disease in Countries Currently
Using Asbestos. Int J Environ Res Public Health.

[11] Terracini B, Pedra F, Otero U (2015). Asbestos-related cancers in Brazil. Cad Saude Publica.

[12] Martin-Chenut K, Saldanha J (2016). O caso do amianto: os limites das soluções locais
para um problema de saúde global. Lua Nova.

[13] Santos SA (2016). Amianto: do uso em larga escala ao banimento. BEPA.

[14] Nolasco LG, Matoso FP, Matos WR (2019). Princípio da precaução para gestão de
riscos do amianto. Rev Dir Pub.

[15] Brasil, Supremo Tribunal Federal (2017). Emenda da Lei Federal 9.055/1995: dispõe sobre a
proibição da extração,
comercialização e exportação do amianto em
território brasileiro.

[16] Porto FA, López-Arranz MA, Fachal FM, Ramos SP (2017). Anuario da Facultade de Ciencias do Traballo da Universidade da
Coruña.

[17] Arantes MD, Cruz NR, Sousa GF, Varoto ACV, Almeida PV (2019). Análise epidemiológica do mesotelioma pleural
maligno no estado de São Paulo, de 2000 a 2015. Braz J Hea Rev.

[18] Salge AKM, Castral TC, Sousa MC, Souza RRG, Minamisava R, Souza SMB (2016). Infecção pelo vírus Zika na
gestação e microcefalia em recém-nascidos:
revisão integrativa de literatura. Rev Eletr Enferm.

[19] Whittemore R, Knafl K (2005). The integrative review: updated methodology. J Adv Nurs.

[20] Moher D, Liberati A, Tetzlaff J, Altman DG, PRISMA Group (2009). Preferred reporting items for systematic reviews and
meta-analyses: the PRISMA statement. PLoS Med.

[21] Wu WT, Lin YJ, Li CY, Tsai PJ, Yang CY, Liou SH (2015). Cancer attributable to asbestos exposure in shipbreaking workers:
a matched-cohort study. PLoS ONE.

[22] Van den Borre L, Deboosere P (2015). Enduring health effects of asbestos use in Belgian industries: a
record-linked cohort study of cause-specific mortality
(2001-2009). BMJ Open.

[23] Chellini E, Martino G, Grillo A, Fedi A, Martini A, Indiani L (2015). Malignant mesotheliomas in textile rag sorters. Ann Occup Hyg.

[24] Kim Y, Myong JP, Lee JK, Kim JS, Kim YK, Jung SH (2015). CT characteristics of pleural plaques related to occupational or
environmental asbestos exposure from South Korean asbestos
mines. Korean J Radiol.

[25] Meisenkothen C (2017). Malignant mesothelioma in a motor vehicle mechanic: case report
and review of the literature. New Solut.

[26] Zu K, Tao G, Goodman JE (2016). Pleural plaques and lung function in the Marysville worker
cohort: a re-analysis. Inhal Toxicol.

[27] Oddone E, Imbriani M (2016). Pleural mesothelioma: case-report of uncommon occupational
asbestos exposure in a small furniture industry. Int J Occup Med Environ Health.

[28] Uguen M, Dewitte JD, Marcorelles P, Loddé B, Pougnet R, Saliou P (2017). Asbestos-related lung cancers: a retrospective clinical and
pathological study. Mol Clin Oncol.

[29] Pira E, Coggiola M, Ciocan C, Romano C, La Vecchia C, Pelucchi C (2017). Mortality of talc miners and millers from Val Chisone, Northern
Italy: an updated cohort study. J Occup Environ Med.

[30] Paris C, Thaon I, Hérin F, Clin B, Lacourt A, Luc A (2017). Occupational asbestos exposure and incidence of colon and rectal
cancers in French men: the Asbestos-Related Diseases Cohort
(ARDCo-Nut). Environ Health Perspect.

[31] Cruz MJ, Sampol J, Pallero M, Rodríguez E, Ferrer J (2018). Asbestos-related disease in upholsterers. Arch Environ Occup Health.

[32] Barbieri PG, Somigliana A, Lombardi S, Festa R, Girelli R, Sarnico M (2017). Mesoteliomi pleurici in addette alla fabbricazione di bambole:
esposizione ad amianto?. Med Lav.

[33] Naik SL, Lewin M, Young R, Dearwent SM, Lee R (2017). Mortality from asbestos-associated disease in Libby, Montana
1979-2011. J Expo Sci Environ Epidemiol.

[34] Carder M, Darnton A, Gittins M, Stocks SJ, Ross D, Barber CM (2017). Chest physician-reported, work-related, long-latency respiratory
disease in Great Britain. Eur Respir J.

[35] Clark KA, Flynn JJ 3rd, Karmaus WJJ, Mohr LC (2017). The effects of pleural plaques on longitudinal lung function in
vermiculite miners of Libby, Montana. Am J Med Sci.

[36] D’Agostin F, De Michieli P, Chermaz C, Negro C (2017). Pleural and peritoneal mesotheliomas in the Friuli Venezia Giulia
register: data analysis from 1995 to 2015 in Northeastern
Italy. J Thorac Dis.

[37] Lee LJH, Lin CK, Pan CH, Cheng Y, Chang YY, Liou SH (2018). Clustering of malignant pleural mesothelioma in asbestos
factories: a subgroup analysis in a 29-year follow-up study to identify
high-risk industries in Taiwan. BMJ Open.

[38] de la Hoz RE, Weber J, Xu D, Doucette JT, Liu X, Carson DA (2019). Chest CT scan findings in World Trade Center
workers. Arch Environ Occup Health.

[39] Barbiero F, Zanin T, Pisa FE, Casetta A, Rosolen V, Giangreco M (2018). Cancer incidence in a cohort of asbestos-exposed workers
undergoing health surveillance. Int Arch Occup Environ Health.

[40] Tamura A, Funakoshi M, Awn JPN, Hasegawa K, Ishimine A, Koike A (2018). Potential asbestos exposure among patients with primary lung
cancer in Japan. J Occup Health.

[41] Park S, Park J, Lee E, Eom H, Shin MY, Kim J (2018). Ovarian cancer in a former asbestos textile factory worker: a
case report. Ann Occup Environ Med.

[42] Rajput Z, Hering KG, Kraus T, Tannapfel A, Sonnenschein G, Centmayer A (2019). Investigating the association between occupational exposure to
asbestos and ovarian carcinoma: results from a pilot study in
Germany. BMC Public Health.

[43] Vimercati L, Cavone D, Caputi A, Delfino MC, De Maria L, Ferri GM (2019). Malignant mesothelioma in construction workers: the Apulia
regional mesothelioma register, Southern Italy. BMC Res Notes.

[44] Takahashi K, Landrigan PJ, Collegium Ramazzini (2016). The global health dimensions of asbestos and asbestos-related
diseases. Ann Glob Health.

[45] Rong Y, Luo X, Zhang Z, Cui X, Liu Y, Chen W (2015). Occupational exposure to asbestos and cardiovascular related
diseases: a meta-analysis. Prev Med Rep.

[46] Kang DM, Kim JE, Kim YK, Lee HH, Kim SY (2018). Occupational burden of asbestos-related diseases in Korea,
1998-2013: asbestosis, mesothelioma, lung cancer, laryngeal cancer, and
ovarian cancer. J Korean Med Sci.

[47] Gogou E, Hatzoglou C, Zarogiannis SG, Malli F, Jagirdar RM, Gourgoulianis KI (2019). Mesothelioma mortality rates in Greece for the period 2005-2015
is increased compared to previous decades. Medicina (Kaunas).

[48] Dragani TA, Colombo F, Pavlisko EN, Roggli VL (2018). Malignant mesothelioma diagnosed at a younger age is associated
with heavier asbestos exposure. Carcinogenesis.

[49] Noonan CW (2017). Environmental asbestos exposure and risk of
mesothelioma. Ann Transl Med.

[50] Ngamwong Y, Tangamornsuksan W, Lohitnavy O, Chaiyakunapruk N, Scholfield CN, Reisfeld B (2015). Additive synergism between asbestos and smoking in lung cancer
risk: a systematic review and meta-analysis. PLoS One.

[51] Merlo DF, Bruzzone M, Bruzzi P, Garrone E, Puntoni R, Maiorana L (2018). Mortality among workers exposed to asbestos at the shipyard of
Genoa, Italy: a 55 years follow-up. Environ Health.

[52] Latifovic L, Villeneuve PJ, Parent MÉ, Kachuri L, Harris SA, Canadian Cancer Registries Epidemiology Group (2020). Silica and asbestos exposure at work and the risk of bladder
cancer in Canadian men: a population-based case-control
study. BMC Cancer.

[53] Luberto F, Ferrante D, Silvestri S, Angelini A, Cuccaro F, Nannavecchia AM (2019). Cumulative asbestos exposure and mortality from asbestos related
diseases in a pooled analysis of 21 asbestos cement cohorts in
Italy. Environ Health.

[54] Korda RJ, Clements MS, Armstrong BK, Law HD, Guiver T, Anderson PR (2017). Risk of cancer associated with residential exposure to asbestos
insulation: a whole-population cohort study. Lancet Public Health.

[55] Marchiori E, Hochhegger B, Zanetti G (2018). Calcificações pleurais. J Bras Pneumol.

[56] Tonet C, Chultz RM, Zimmer MF, Silva NO (2019). Relação entre pneumoconioses e o câncer de
pulmão. Rev Uninga.

[57] Silva CCS, Rodrigues LMC, Silva VKBA, Silva ACO, Silva VLA, Martins MO (2013). Percepção da enfermagem sobre
condições de trabalho em unidades de saúde da
família na Paraíba - Brasil. Rev Eletr Enferm.

